# Investigating Latent Interactions between Students’ Affective Cognition and Learning Performance: Meta-Analysis of Affective and Cognitive Factors

**DOI:** 10.3390/bs13070555

**Published:** 2023-07-04

**Authors:** Jian Li, Eryong Xue, Chenchang Li, Yunshu He

**Affiliations:** China Institute of Education Policy, Faculty of Education, Beijing Normal University, Beijing 100875, China; jianli209@bnu.edu.cn (J.L.);

**Keywords:** affective and cognitive factors, student learning performance, education development, student learning development

## Abstract

Affective and cognitive factors play significant roles in influencing students’ learning performance. However, limited studies exist that examine the latent interactions between these factors and students’ learning performance. This study applied a meta-analytic approach to examine the relationships between affective and cognitive factors and students’ learning performance through the selected publications. We identified 18 affective and cognitive influencing factors related to student learning achievement/performance. It was found that academic performance was significantly impacted by learning scores, future aspirations and goals, peer support for learning, and family support for learning. A moderate impact was observed for cognitive benefits, skill development, self-regulation, values, knowledge, character, self-belief, attitudes and beliefs, affective benefits, motivation, optimism, and behavioral engagement. A weak influence was observed for control and relevance of schoolwork and self-efficacy. The discussion and limitations of this study have also been provided in the last sections.

## 1. Introduction 

Affect and cognition are important factors that influence students’ academic performance. According to the theory of aptitude, the measurement of aptitude includes not only cognitive factors (i.e., motivation) but also emotional factors (i.e., emotion) that affect students’ academic performance [1,2,3,4,5,6]. Increasingly, more scholars are paying attention to the influence of students’ emotions on their academic achievement. Cognition and emotion can directly affect students’ academic performance [7,8,9,10]. For example, Bachtiar (2010) integrated emotional factors and cognitive factors in a comprehensive evaluation of cognitive reappraisal ability (CRA). CRA is often used to assess English learning achievement using fuzzy inference. The results suggested that CRA-based FIS is useful for assessing student performance using emotional and cognitive factors to present concrete outcomes. Heikkila et al. (2012) explored the happiness and academic achievement of normal students, focusing on the influence of motivation and emotional factors on students’ learning. The results showed that students with high motivation and high norms (cognitive and emotional factors) had high academic achievement and reported high overall happiness [11,12]. 

Affective and cognitive factors have been identified as crucial elements that significantly influence students’ learning performance [13,14,15]. However, few studies have examined interactions between affective and cognitive factors and students’ learning performance. This study aims to address the significant problem of exploring the latent interaction between students’ affective cognition and learning performance. The research questions are provided as follows: 

Q1: What is the latent interaction between students’ affective cognition and learning performance?

Q2: How can we identify the affective and cognitive influencing factors related to students’ learning achievement/performance? 

Therefore, we conducted a meta-analysis to examine relationships between those factors and students’ learning performance. Along with the research questions above, this paper is divided into several sections: Section 1 explores a literature review on the influence of affective and cognitive factors on students’ academic performance and the influence of cognition and emotion on students’ academic performance. The method is provided in Section 2, which includes research methods and data sources, document coding and processing, and a publication bias test. The result sections include overall effect-value analysis, specific effect-value analysis, a heterogeneity test, and publication bias analysis and effect-value analysis. The discussion, limitations, implications, and conclusion are offered in Section 6. The intended audience of this study may focus on exploring the latent interaction between students’ affective cognition and learning performance, as well as identifying the affective and cognitive influencing factors related to students’ learning achievement/performance. Additionally, the intended audience will benefit from examining the latent interactions between these factors and students’ learning performance.

### 1.1. Literature Review on the Influence of Affective and Cognitive Factors on Students’ Academic Performance

Different scholars offer various approaches to investigating the influence of affective and cognitive factors on students’ academic performance [16]. For example, Kim et al., (2014) used a three-step hierarchical multiple regression method to explore the effects of motivation (i.e., self-efficacy and intrinsic value), math achievement emotion (i.e., anxiety, anger, shame, despair, boredom, enjoyment, and pride), and cognitive processes (i.e., use of cognitive strategies and self-regulation) on students’ math scores. The results showed that motivation accounted for about 13% of the variance in scores. Self-efficacy is an important individual predictor of student achievement. However, when achievement emotion was added to the analysis, self-efficacy did not predict student achievement, and emotion accounted for 37% of the variance in achievement. The use of cognitive strategies and self-regulation did not explain any additional differences in final scores. Eichler and Gradwohl (2021) explored the supportive, emotional, motivational, and cognitive factors that influence the math achievements of the first-year engineering students. The results showed that students’ mathematical ability before the first semester and the tutoring support they received during the first semester were the factors contributing to their success in the final exam of their first year of engineering studies. With the continuous expansion of higher education, the teacher–student ratio is gradually decreasing, and teachers are facing increasing demands for student guidance. This can lead to a weakening of the emotional connections between teachers and students. Loon and Bell (2018) explored the importance of emotions in improving students’ cognitive skills and how emotions interact with knowledge and reflection [17]. Their research results showed that emotions regulated the direct relationship between knowledge and cognitive skills through reflection, as well as the indirect relationship between knowledge and cognitive skills, and that emotions play a crucial role in students’ learning. Sæle et al., (2016) investigated the psychometric properties of a new literacy problem (LP) scale and its impact on grade point average (GPA). Their analysis demonstrated that the scale exhibits good psychometric characteristics. Furthermore, they found that learning trajectory choice, task-solving skills, gender, lack of an education plan, and LP scores predicted GPA in descending order. Using a questionnaire survey, Sayani et al., (2019) investigated students’ perceptions and experiences of emotional, cognitive, and skill development, and assessed whether those perceptions and experiences affected students’ participation in case competitions. The results revealed statistically significant differences in students’ cognitive and emotional gains between perceived learning and actual learning. Moreover, students’ perspectives on learning and real-life experience were significantly and positively correlated with their participation in the case-study competitions. Song and Wang (2021) examined the extent to which disciplinary, cognitive, and affective factors can explain individual differences in students’ interdisciplinary abilities. Multiple linear regression analysis showed that students’ subject knowledge (DK), attitudes toward interdisciplinary approaches, and interdisciplinary learning opportunities were all statistically significant predictors in the added predictive models. The results from semi-structured interviews indicated that students did not demonstrate substantial engineering design knowledge in their responses [4]. Furthermore, the study found that students’ perceptions of interdisciplinary learning influenced their application of subject knowledge (DK) to generate comprehensive insights into various aspects related to possible solutions. When learners have good cognitive and emotional abilities, they will correspondingly achieve good academic performance. However, Suldo et al., (2018) examined the effects of 34 factors on AP and IB student achievement and found that good mental health and academic achievement were associated with higher levels of achievement motivation and cognitive engagement, as well as lower levels of parent–child conflict, stress from major life events, and the use of avoidance coping strategies. Higher levels of emotional engagement, the use of coping methods, and parents who parented with scientific authority were strong predictors of positive mental health outcomes but were not associated with academic outcomes [18,19].

### 1.2. The Influence of Cognition and Emotion on Students’ Academic Performance in Different Internet Learning Modes

In the era of mobile learning and ubiquitous learning, it is of great practical significance to examine the influence of cognition and emotion on students’ academic performance [20,21]. In one such study, Kongcharoen et al. took online social network (OSN) attention as a moderating factor to study the impact of affective and cognitive factors on learning achievement (LA). The results confirmed that student engagement and LA differed significantly across the whole group, while student engagement, emotional scores, and LA were significantly different in the high OSN attention group. No significant differences were observed between the low OSN attention groups. Furthermore, for students with higher rather than lower OSN attention, academic performance can better promote the impact of emotional and cognitive levels. The flipped classroom model has a significant effect on improving learners’ participation and active learning results. Only content/learning-related outcomes, not general ability or satisfaction were influenced by academic ability and epistemological beliefs. Both pre-class and in-class participation levels had an impact on most types of foreign-language achievement, except for general ability, which was only affected by in-class participation level. Learners’ emotional engagement was not affected by epistemological beliefs but had direct and indirect effects on foreign-language learning outcomes through behavioral and cognitive engagement [22,23,24]. The intervention of factors that affect students’ academic performance through technology can also improve students’ academic performance to a certain extent. Hong et al., (2017) designed a prediction-observation-interpretation-based scientific inquiry learning model and implemented it in an electronic application called “Why”. They analyzed the effects of using “Why” on students’ interest in learning science (ILS), cognitive anxiety (CA), and external cognitive load (ECL) through confirmatory factors of structural equation models. The results demonstrated that students with high ILS exhibited low CA and ECL. Following the utilization of “Why” to establish connections, students experienced a significant improvement in self-confidence, which subsequently led to enhanced academic performance [25,26].

### 1.3. Impacts on Students’ Achievement using Cognition and Affection as the Components of Students’ Engagement

Cognition and emotion, as components of students’ engagement, have been found to impact academic performance [17,27,28,29,30]. Student participation is a significant factor that influences academic performance and serves as the foundation for dropout prevention and high school reform measures. Several scholars have developed student participation scales that assess cognitive, emotional, and other factors, which indirectly reflect students’ academic performance. The Student Engagement Tool (SET), for example, was designed to measure two components of student engagement: cognitive and emotional engagement. Betts et al. verified in their study that the SET can effectively gauge the cognitive and emotional participation of students at the middle and high school levels. However, many existing student engagement scales primarily measure broader school-level participation rather than specific class- or student-level participation [31].

To gain a deeper understanding and measure student participation at the micro level, Whitney et al., (2019) developed a statistical scale that encompasses three aspects: emotional, behavioral, and cognitive characteristics. Additionally, they demonstrated the scale’s practicability in predicting academic performance, presenting specific results. McGeown et al. created a mental resilience scale for adolescents that includes challenges, commitment, confidence (abilities and interpersonal relationships), and control (life and emotions). The study also found that the psychological resilience attributes of adolescents were associated with their learning motivation and engagement, happiness (depression and anxiety), test anxiety, as well as other emotional, cognitive, and behavioral tendencies, thus impacting their academic performance and mental health. Following the SOR theory of behaviorism, Yang and Cheng (2019) investigated factors that affect the persistence of college students’ mobile learning from two aspects: self-determination needs and learning engagement [32,33,34]. The results showed that perceived learning support, learning self-management, and peer influence significantly influenced affective learning participation and cognitive learning participation. As a result, these factors positively impacted sustained intentions to use mobile learning [35,36,37].

## 2. Methods

### 2.1. Research Methods and Data Sources

As a “re-statistics” method, meta-analysis provides a statistically effective approach to literature research, distinct from the qualitative descriptions and summaries typically in literature reviews. Following common meta-analytic methods, we conducted a comprehensive examination of different empirical studies, calculated the average effects, integrated the literature, performed secondary statistical analysis on the data, and obtained the real effects of different influencing factors. In addition, we derived extensive and effective conclusions and implications in the end. In accordance with widely accepted meta-analysis methodology, we performed the following steps: (1) literature collection and screening, focusing on quantitative empirical studies in line with the research topic; (2) literature coding and formulation of coding rules to describe the basic information extracted from the relevant literature; and (3) statistical testing and analysis, calculation of effect size, and heterogeneity analysis.

Because the reliability of meta-analysis results depends on the comprehensiveness and validity of the literature collected, we conducted an extensive search of all the literature published in English between 2000 and 2022. In accordance with the theme of this study, we used the search terms, “education”, “affective and cognitive factors”, and “student”. A total of 592 relevant publications were retrieved from the English database Web of Science, which were then narrowed down to education-related subtopics, resulting in 453 eligible publications.

To ensure a scientific and rational literature-screening process, we specified screening rules to obtain relevant studies that were consistent with our theme and to produce data that could be statistically analyzed. The criteria for literature selection were as follows: (1) The topic of the literature research must be related to the impact of emotional and cognitive factors on students’ academic performance, and the dependent variable must be a relevant measurement index of students’ academic success (e.g., academic performance or personal achievement); (2) the literature must be empirical research, excluding qualitative research results; (3) there can be no duplication in the literature, that is, multiple articles with the same research sample by the same author; (4) studies must report statistical measures of relevant influencing factors, including sample size, Pearson correlation coefficient, or other effect sizes that can be translated into the correlation coefficient (e.g., t-value, *p*-value, or F-value).

**Figure 1:**
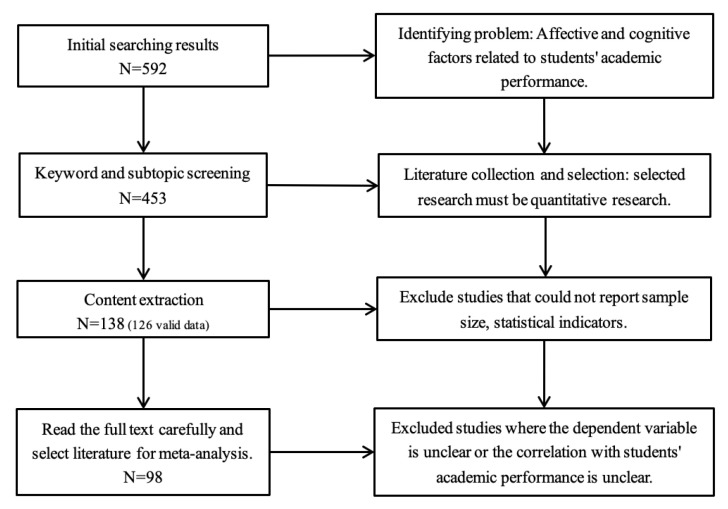
Publication screening process.

The article selection process consisted of several stages. In the initial stage of searching, we used keywords related to affective and cognitive factors in relation to students’ academic performance. During this stage, 592 publications were selected based on their relevance to research themes. In the second stage of keywords and subtopic screening, among the selected 592 publications, we re-selected the key samples and deleted the non-quantitative studies, and at last, 453 publications were selected to meet the method criteria. In the third stage, we excluded the irrelevant publications that could not report the sample size and accurate statistical indicators. In the content extraction stage, 138 selected samples were selected. Among them, 12 (6.42%) were related to the research topic but did not report data. By the end, we eliminated studies with unclear dependent variables or unclear correlations between dependent variables and students’ academic studies, and a total of 98 publications were finally selected for the meta-analysis (See Figure 1).

### 2.2. Document Coding and Processing

After selecting eligible publications for the meta-analysis, the collected literature must be coded, which facilitates the identification of subsequent influencing factors, related effect sizes, and sample sizes. In this study, we recorded information including author, title, publication year, sample size, effect size category, effect size value, and influencing factors in each article. Considering that Pearson correlation coefficient has been widely used in the social sciences and is the preferred effect value for meta-analysis in education measurement, we chose the correlation coefficient as the effect size metric for this study. For publications in which no correlation coefficient is reported, but the sample size and t-value, F-value, and $\chi^{2}$-value are reported, the correlation coefficient can be uniformly calculated using the following formula, where df is the degree of freedom:

$$r=\sqrt{\frac{t^{2}}{\left(t^{2}+df\right)}}=\sqrt{\frac{F\cdot df_{1}}{F\cdot df_{1}+df_{2}}}=\sqrt{\frac{\chi^{2}}{N(k-1)}}$$

In addition, to distinguish the impact on students’ studies of emotional and cognitive factors, we divided the influencing factors into three categories: (1) emotional, (2) cognitive, and (3) behavioral. Table 1 presents examples of document coding.

### 2.3. Experimental Design and Data Analysis

In this study, we used R (v4.2.0), a meta-analysis package, to analyze the data. All model parameters were estimated using the restricted maximum likelihood method, and results with a two-tailed *p*-value less than 0.05 were defined as significant. It was assumed that the studies included in the meta-analysis were independent of each other and that different publications did not influence one another. Among the 98 studies included in the meta-analysis, a total of 93 influencing factors were identified that affected students’ academic performance. Considering that some factors with different names have the same meaning, the variables with similar meanings were uniformly named as the variables that occur most frequently before conducting the actual analysis. After summarizing and combining factors, a total of 42 cognitive and emotional factors that reportedly had an impact on students’ academic performance were retained. Given the need to ensure the reliability of the meta-analysis, this paper provides only the influential factors that were reported in at least in two studies. Table 2 shows the comprehensive effects of some influencing factors on students’ academic performance, as reported in the analyzed literature. The effects reflect the correlation between the factors and students’ academic performance, and whether those effects were positive or negative. Except for anxiety, all factors had a positive effect on students’ academic development. Table 2 also presents the 95% confidence interval of comprehensive correlation coefficients for each influencing factor, along with the significance of the two-tailed test, and Rosenthal’s fail-safe number for publication bias test. Heterogeneity test results for each influencing factor are reported in Table 3.

### 2.4. Publication Bias Test

Some studies have indicated the presence of publication bias in meta-analyses due to the higher likelihood of publishing studies with statistically significant findings. Publication bias can introduce bias in effect size estimates, ultimately leading to less-accurate results of a meta-analysis. Thus, the true effect of a certain influencing factor cannot be sufficiently estimated. In general, when publication bias is present, the meta-analysis results tend to overestimate the true extent of an impact factor. Qualitative methods, such as funnel plots, can be employed to assess the presence of publication bias. When the funnel plot is basically symmetrical, it indicates that publication bias can be ignored; otherwise, it is necessary to revise the included literature. However, funnel plots are a more subjective judgment method, while Egger’s regression test and Rosenthal’s fail-safe number—used in this study—can be referred to as more objective methods.

## 3. Results

### 3.1. Overall Effect-Value Analysis

**Figure 2:**
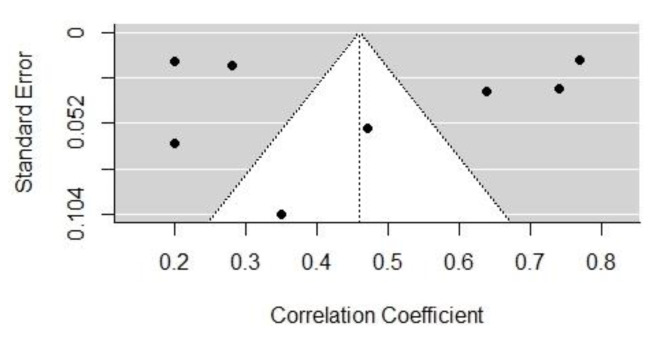
The funnel plot of the effect size of cognitive dominance.

**Figure 3:**
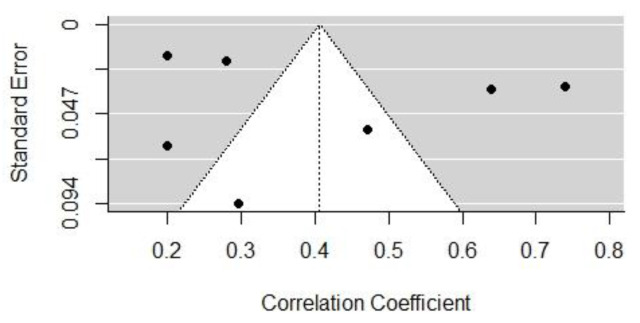
The funnel plot of the effect size of emotional dominance.

Figure 2 and Figure 3 show the funnel plots of the effect sizes of the two influencing factors, cognitive dominance, and emotional dominance, respectively. According to the image analysis, the funnel plots were basically symmetrical and, thus, there was no obvious publication bias. However, since the funnel plot is a more subjective judgment method that may not always provide consistent reliability, this study employed a combination of the Egger’s regression test and the assessment of publication bias using Rosenthal’s fail-safe number. The black dots mean the correlated distribution of the selected sample. 

Taking studies of the above two influential factors (cognitive and emotional dominance) as an example, under the mixed-effects model, the *p*-values of Egger’s regression tests were 0.5094 and 0.7392, respectively, indicating that there was insufficient evidence to reject the non-existent publication bias hypothesis. Therefore, it can be concluded that there was no discernible publication bias in studies of those two factors. In studies of most other influential factors, Egger’s test also indicated that no obvious publication bias was present. Egger’s test requires a minimum number of studies that include a certain influential factor, so it is not applicable to a publication bias test that includes only two studies. Therefore, we further investigated the publication bias of influential factors using Rosenthal’s fail-safe number.

### 3.2. Specific Effect-Value Analysis

Rosenthal’s fail-safe number determines the existence of publication bias by calculating how many missed studies must be added to make statistically significant meta-analysis results statistically insignificant. When the fail-safe number is N < 5k + 10, it indicates that the results of the meta-analysis may be affected by publication bias, and the results of the meta-analysis are therefore unreliable, where k represents the number of studies included in a certain impression factor. Table 2 also lists the publication bias test results for some influencing factors; the test results were calculated using Rosenthal’s fail-safe number. There was no publication bias for most of the influencing factors, and only the fail-safe numbers for Interest, Task Management, and Anxiety were less than 5k + 10, which indicated serious publication bias. The results of the meta-analysis were not convincing and should be rejected in the subsequent analysis.

### 3.3. Heterogeneity Test and Publication Bias Analysis

Heterogeneity measures the size of variation among studies on the same topic. When the true effects of all selected studies are the same, heterogeneity is 0 and a fixed-effects model should be used. By contrast, if the combined effects of different studies are different, the heterogeneity between studies cannot be ignored, and a random-effects model should be adopted. Most meta-analyses use either a fixed-effects model or a random-effects model. In the case of fixed effects, all studies are assumed to have the same real effect, while random effects allow different studies to have different real effects, and the real effects obey the distribution of some mean. Under different model assumptions, the comprehensive effect estimation methods are different. For fixed-effects models, the weight of each study is the inverse of the variance of each study, and the combined effect is the weighted average of each study.

$$W_{i}=\frac{1}{V_{Y_{i}}}$$

$$M=\frac{\sum_{i=1}^{k}W_{i}Y_{i}}{\sum_{i=1}^{k}W_{i}}$$

The random-effects model assumes that the observed effect size consists of the total mean, the deviation between the studied true effect size and the total mean, and the deviation between the observed effect size and the true effect size.

$$Y_{i}=\mu +\zeta_{i}+\varepsilon_{i}$$

Thus, for a random-effects model, we need to know not only the variance within the study, but also the variance between the studies. In the present study, the variance between studies was estimated using the moment method. The estimated variance between studies was calculated as:

$$W^{*}{}_{i}=\frac{1}{V_{Y_{i}}{}^{*}}$$

$$V_{Y_{i}}{}^{*}=V_{Y_{i}}+T^{2}$$

$$M=\frac{\sum_{i=1}^{k}W_{i}{}^{*}Y_{i}}{\sum_{i=1}^{k}W_{i}{}^{*}}$$

To determine which model should be used in our meta-analysis, we first conducted a heterogeneity test. There are many methods for testing heterogeneity, but the most frequently used are Q tests. Using the Q statistic, we calculated the weighted sum of squares of the deviation from the average effect for each study, which followed a chi-square distribution. When the *p*-value of the Q test was less than 0.05, we determined that there was obvious heterogeneity between studies, and it was more reasonable to adopt the random-effects model; otherwise, the fixed-effects model was adopted. This method was used to judge whether there was heterogeneity in a study that was included because of an influential factor, and how much heterogeneity was needed to be measured statistically.

As illustrated in Table 3, except for the three influential factors, Knowledge, Self-Efficacy, and Interest, the heterogeneity tests of other influential factors all reached the level of significance (*p* < 0.05), and most of the influential factors exhibiting heterogeneity were greater than 75%. Therefore, we used a random-effects model to estimate the comprehensive effect size of each influential factor, except for Knowledge, Self-Efficacy, and Interest.

### 3.4. Effect-Value Analysis

According to the results of the heterogeneity test, a random-effects model was used to combine 18 influencing factors with heterogeneity, while a fixed-effects model was used to combine 3 influencing factors without heterogeneity (Knowledge, Self-Efficacy, and Interest). The combined effect is presented in Table 2. Table 2 also presents the 95% confidence interval of the combined effect and the *p*-value of the two-tailed test, which not only determined the strength of the combined effect of a certain research factor, but also showed the reliability of the estimated results. To classify the degree of influence of different influencing factors on students’ academic performance, we selected the classification criteria for an effect size proposed by J. Cohen: r < 0.1 means basically no correlation, 0.1 ≤ r < 0.3 means weak correlation, 0.3 ≤ r < 0.5 means medium-strength correlation, and r ≥ 0.5 means strong correlation. According to the double-tail test and publication bias results for each influencing factor, three factors (Interest, Task Management, and Anxiety) were excluded, as they failed the publication bias test. Table 4 lists 18 influencing factors that had varying degrees of influence on students’ academic performance.

## 4. Discussion

This study applied a meta-analytic approach to investigate the relationships between affective and cognitive factors in student learning performance [38,39,40,41,42,43,44]. Our analysis finally identified 18 influencing factors related to student learning achievement/performance. We found that Learning Scores, Future Aspirations and Goals, Peer Support for Learning, and Family Support for Learning had a significant impact on students’ academic performance. Cognitive Benefits, Skill Development, Self-Regulation, Values, Knowledge, Character, Self-Belief, Attitudes and Beliefs, Affective Benefits, Motivation, Optimism, and Behavioral Engagement had a moderate impact on students’ academic performance. Control and Relevance of School Work and Self-Efficacy had a weak influence on students’ performance. Among the factors that significantly impacted students’ academic performance, the cognitive factors comprised Learning Scores and Future Aspirations and Goals, which is consistent with general research and cognition [45,46,47,48]. Academic achievement and personal goals for the future had a significant positive effect on students’ academic achievement. The emotional factors, Peer Support for Learning and Family Support for Learning, had important supporting effects on students’ academic performance. Creating a good peer learning atmosphere and family education atmosphere is of great emotional significance to students’ individual academic development [49,50,51,52,53]. Among the factors that had a moderate impact on student achievement, the cognitive factors were Cognitive Benefits, Skill Development, Self-Regulation, Values, Knowledge, and Character. These cognitive adjustments indicate that they also have a good intervention effect on students’ academic development. Furthermore, Self-Belief, Attitudes and Beliefs, Affective Benefits, Motivation, and Optimism indicated positive effects on students’ academic performance from the aspects of emotion and attitude. By contrast, the cognitive factor, Control and Relevance of School Work, and the emotional factor, Self-Efficacy, had little importance in students’ academic efficacy and were not considered the primary factors for improving students’ academic ability [54,55,56,57].

Many factors affect students’ academic performance, including internal and external motivation, self-efficacy, and self-initiative, among others [58,59,60,61,62,63]. Scholars have also explored the effects of cognitive and emotional components on these factors. For example, Arpaci and Basol (2020) examined the impact of preservice teachers’ cognitive and technological perceptions of their continuous intention to use the flipped classroom model. They found that both self-regulation and self-efficacy have a positive impact on perceived ease of use. The perceived anxiety has a negative impact on self-efficacy, and self-efficacy mediates the relationship between perceived anxiety. They also suggested that there are significant relationships between cognitive and technological factors and continuous intention to use the flipped classroom model. In addition, Hammoudi (2019) examined the relationship between students’ motivation to succeed in an introductory mathematics course (as the dependent variable of the study) offered by a university in the United Arab Emirates and five other independent variables (i.e., cognitive mathematical self-concept, emotional mathematical self-concept, extrinsic motivation such as expectations of future career and income, the age of the students, and the number of mathematics courses taken by the students). The quantitative correlation observed between student motivation and these five variables showed a theoretically consistent interrelationship, while the five independent variables explained 71.3% of the change in students’ motivation for success. Initiative is the intrinsic ability of students to regulate, control, and supervise their own learning [64,65,66,67,68,69].

The effectiveness of learners at regulating their cognitive, emotional, and behavioral processes as they interact in a learning environment is critical to their academic success. Code proposed a theory of learner agency, or agency for learning (AFL), which is an emergent ability to intentionally self-generate and respond to social factors in a learning environment [70,71,72,73,74].Code further explored the predictive validity and psychometric properties of the agency for learning questionnaire (AFLQ), which covers four dimensions of agentic function, including intentionality (planning, decision-making ability), forethought (intrinsic and extrinsic motivation), self-regulation, and self-efficacy. The research results showed that “the AFLQ provides a reliable, valid, multidimensional measure of AFL based on existing theoretical and empirical findings”. Overall, good cognitive ability is generally positively correlated with students’ academic performance, while most studies report that emotional ability also directly affects students’ academic performance. Additionally, students’ emotional ability often does not have a direct impact on their academic performance, but instead affects it indirectly through emotional factors such as attitude, motivation, and sense of efficacy. In the era of the Internet, furthermore, it is also worth exploring how students’ academic performance is affected by the new learning or teaching modes that have emerged online [75,76,77]. 

The findings of the present study provide a relatively comprehensive estimate of the reported relationships between affective and cognitive factors and students’ learning performance in international empirical studies. The study offers a systematic exploration of the implicit interactions among affective and cognitive factors in students’ learning performance. Moreover, this meta-analysis elucidates teaching research that is focused on the promotion of affective and cognitive factors to improve students’ learning performance.

## 5. Limitations

Some limitations of the present study should be noted. The literature examining the relationships between affective and cognitive factors and students’ learning performance should include more relevant research. Furthermore, the research topic could be extended to include additional relevant themes, such as internal or external affective and cognitive actions, and implicit or explicit affective and cognitive actions. In addition, we also suggested that future researchers could add more psychology points of view that deepen the interaction between students’ affective cognition and learning performance. For example, in future research, differences and similarities in the relationships between affective and cognitive factors and students’ learning performance should be studied more extensively, together with moderating variables, to derive more convincing universal conclusions.

## 6. Implications and Conclusions

This study contributes to research that applies a meta-analysis approach to examining relationships between affective and cognitive factors in student learning performance. Of the 18 influencing factors identified, we found that Learning Scores, Future Aspirations and Goals, Peer Support for Learning, and Family Support for Learning have the strongest impact on students’ academic performance. This study applied a meta-analytic approach to examine the relationships between affective and cognitive factors and students’ learning performance through the selected publications. In total, the 18 affective and cognitive influencing factors related to student learning achievement/performance were selected and we found that academic performance was significantly impacted by Learning Scores, Future Aspirations and Goals, Peer Support for Learning, and Family Support for Learning. A moderate impact was observed for Cognitive Benefits, Skill Development, Self-Regulation, Values, Knowledge, Character, Self-Belief, Attitudes and Beliefs, Affective Benefits, Motivation, Optimism, and Behavioral Engagement.

## Figures and Tables

**Table 1:** Affective and cognitive factors affecting students’ academic performance: examples from the literature.

No.	Authors	Year	Sample Size	Effect Size Class	Influencing Factor
1	Joseph E. Betts	2010	2416	correlation coefficient	(1) Teacher–student relationships (0.74)
					(2) Control and relevance of school work (0.7)
					(1) Peer support for learning (0.76)
					(2) Future aspirations and goals (0.80)
					(1) Family support for learning (0.79)
2	Fitra A. Bachtiar	2015	188	correlation coefficient	(1) Motivation (0.526)
					(1) Introversion (−0.145)
					(1) Extroversion (0.677)
					(1) Anxiety (−0.304)
					(2) Learning scores (0.603)
3	Hameedah Sayani	2018	204	t-value	(2) Cognitive benefits (7.62)
					(2) Skill development (2.36)
					(1) Affective benefits (4.95)
4	Brendan M. Whitney	2018	195	correlation coefficient	(2) Cognitive benefits (0.74)
					(1) Affective benefits (0.73)
					(3) Behavioral benefits (0.76)
5	Sarah McGeown	2016	439	correlation coefficient	(2) Challenge (0.59)
					(1) Interpersonal confidence (0.21)
					(1) Confidence in abilities (0.46)
					(1) Control of emotions (0.24)
					(2) Control of life (0.43)
					(1) Commitment (0.31)
					(1) Self-belief (0.74)
					(1) Persistence (0.6)
					(2) Valuing (0.79)
					(2) Task management (0.55)
					(2) Planning (0.57)
					(1) Anxiety (0.27)

**Table 2:** Results of the meta-analysis based on comprehensive correlation coefficients.

Influencing Factor	K	N	R	95% Confidence Interval		Z Test *p*-Value	Fail-Safe Number
				Lower Limit of Interval	Upper Limit of Interval		
Learning Scores	2	710	0.6591	0.5653	0.7530	0.0001	317
Future Aspirations and Goals	4	4596	0.5561	0.1498	0.2625	0.0002	3086
Peer Support for Learning	3	5104	0.5529	0.2784	0.8274	0.0001	2019
Family Support for Learning	2	4795	0.5103	0.0595	0.9611	0.0265	1361
Self-Belief	3	1155	0.4688	0.113	0.8246	0.0098	452
Cognitive Benefits	8	7495	0.4596	0.2937	0.6255	0.0001	3139
Skill Development	2	710	0.4354	−0.0908	0.9616	0.1049	174
Attitudes and Beliefs	4	1540	0.4305	0.167	0.694	0.0014	628
Affective Benefits	7	6834	0.4066	0.2427	0.5704	0.0001	1581
Motivation	2	2567	0.398	0.1572	0.6389	0.0012	177
Self-Regulation	3	1437	0.3706	0.0642	0.677	0.0178	149
Behavioral Engagement	4	6225	0.356	0.1013	0.6107	0.0062	652
Values	3	721	0.3523	0.0651	0.6395	0.0162	126
Optimism Emotion	5	2522	0.3522	0.2403	0.4641	0.0001	451
Knowledge	2	546	0.3423	0.2577	0.4269	0.0001	48
Character	2	1244	0.3212	−0.0757	0.718	0.1127	51
Control and Relevance of School Work	3	6455	0.2069	−0.4422	0.8559	0.5322	696
Self-Efficacy	4	1490	0.1354	0.0856	0.1853	0.0001	36
Interest	2	362	0.1242	0.0225	0.2258	0.0167	3
Task Management	2	2099	0.0947	−0.8755	1.0649	0.8482	2
Anxiety	4	851	−0.1249	−0.4043	0.1545	0.3809	1

**Table 3:** Heterogeneity test results of the meta-analysis.

Influencing Factor	K	N	Heterogeneity (Q Test)					
			Q-Value	*p*-Value	$I^{2}$	$\tau^{2}$	τ	SE
Learning Scores	2	710	3.7485	0.0529	73.32%	0.0034	0.0587	0.1425
Future Aspirations and Goals	4	4596	415.8226	0.0001	99.64%	0.0892	0.2986	0.1498
Peer Support for Learning	3	5104	478.9595	0.0001	99.42%	0.0583	0.2415	0.1401
Family Support for Learning	2	4795	478.8238	0.0001	99.79%	0.1056	0.3249	0.23
Self-Belief	3	1155	70.5584	0.0001	98.77%	0.0968	0.3112	0.1815
Cognitive Benefits	8	7495	856.6447	0.0001	98.75%	0.0549	0.2343	0.0846
Skill Development	2	710	55.6388	0.0001	98.20%	0.1416	0.3763	0.2685
Attitudes and Beliefs	4	1540	111.0299	0.0001	98.15%	0.0703	0.2651	0.1345
Affective Benefits	7	6834	323.7459	0.0001	98.03%	0.0464	0.2154	0.0836
Motivation	2	2567	19.1809	0.0001	94.79%	0.0287	0.1694	0.1229
Self-Regulation	3	1437	130.4481	0.0001	97.45%	0.0693	0.2632	0.1563
Behavioral Engagement	4	6225	224.0673	0.0001	99.06%	0.0662	0.2572	0.1299
Values	3	721	44.2021	0.0001	93.68%	0.0592	0.2432	0.1465
Optimism Emotion	5	2522	1299.8517	0.0001	99.12%	0.1653	0.4065	0.0571
Knowledge	2	546	1.239	0.2657	19.29%	0.0008	0.0279	0.0432
Character	2	1244	44.1145	0.0001	97.73%	0.0802	0.2831	0.2025
Control and Relevance of School Work	3	6455	2719.05	0.0001	99.92%	0.3287	0.5733	0.3311
Self-Efficacy	4	1490	4.7646	0.1899	0.12%	0	0.0022	0.0254
Interest	2	362	0.2459	0.62	0.00%	0	0	0.0519
Task Management	2	2099	702.2907	0.0001	99.86%	0.4894	0.6995	0.495
Anxiety	4	851	28.9496	0.0001	91.14%	0.0346	0.1859	0.0993

**Table 4:** Influencing factors related to students’ academic performances.

Correlation Coefficient		Influencing Factors
Strong correlation (r ≥ 0.5)	Cognitive Factor	Learning Scores (0.6591), Future Aspirations, and Goals (0.5561)
	Affective Factor	Peer Support for Learning (0.5529), Family Support for Learning (0.5103)
Medium correlation (0.3 ≤ r ≤ 0.5)	Cognitive Factor	Cognitive Benefits (0.4596), Skill Development (0.4354), Self-Regulation (0.3706), Values (0.3523), Knowledge (0.3423), Character (0.3212)
	Affective Factor	Self-Belief (0.4688), Attitudes and Beliefs (0.4305), Affective Benefits (0.4066), Motivation (0.398), Optimism Emotion (0.3522)
	Behavioral Factor	Behavioral Engagement (0.356)
Weak correlation (0.1 ≤ r ≤ 0.3)	Cognitive Factor	Control and Relevance of School Work (0.2069)
	Affective Factor	Self-Efficacy (0.1354)

## Data Availability

Not applicable.
